# MFAP4 is a novel prognostic biomarker in glioma correlating with immunotherapy resistance and ferroptosis

**DOI:** 10.3389/fphar.2025.1551863

**Published:** 2025-02-21

**Authors:** Yuanhao Lv, Ying Gao, Wenyu Di, Zhaoyi Li, Yashen Shi, Tianyuan Hou, Yiyang Chen, Jiaqi Tian, Miaoming Xu, Wei Su, Min Zhang, Jiateng Zhong

**Affiliations:** ^1^ Department of Pathology, The First Affiliated Hospital of Xinxiang Medical University, Xinxiang, China; ^2^ Department of Pathology, School of Basic Medical Sciences, Xinxiang Medical University, Xinxiang, China; ^3^ Department of Pediatric, Shidong Hospital, Shidong Hospital Affiliated to University of Shanghai for Science and Technology, Shanghai, China; ^4^ Xinxiang Engineering Technology Research Center of Digestive Tumor Molecular Diagnosis, The First Affiliated Hospital of Xinxiang Medical University, Xinxiang, China; ^5^ Department of Oncology, The First Affiliated Hospital of Xinxiang Medical University, Xinxiang, China

**Keywords:** MFAP4, prognosis, biomarker, immunotherapy, gliomas, ferroptosis

## Abstract

**Background:**

Glioma, an aggressive brain tumor, poses a challenge in understanding the mechanisms of treatment resistance, despite promising results from immunotherapy.

**Methods:**

Genes associated with immunotherapy resistance were identified by analyzing The Cancer Genome Atlas (TCGA), the Chinese Glioma Genome Atlas (CGGA), and the Gene Expression Omnibus (GEO) database. In addition, gene set enrichment analysis (GSEA) was utilized to reveal relevant signaling pathways. Co-expression, differential expression and functional analyses were performed using TCGA-GBM/LGG, TIMER 2.0, MetScape, GTEx and LinkedOmics databases. Relationships with immune infiltration, ferroptosis and immune checkpoint genes were assessed. Gene mutations were explored by cBioPortal. Logistic regression, Lasso analysis, Receiver Operating Characteristic (ROC), Kaplan-Meier analysis, and Nomogram modeling assessed the correlation between MFAP4 and clinicopathological features of gliomas. By analyzing different datasets, we found that MFAP4 was aberrantly overexpressed in gliomas and correlated with poor clinicopathological features of gliomas. MFAP4 was an independent prognostic indicator and significantly correlated with glioma progression. We also performed functional and pathway enrichment analyses of MFAP4 in gliomas to explore its biological functions and potential molecular mechanisms in gliomas.

**Results:**

MFAP4 was significantly elevated in glioma tissues compared to controls. MFAP4-related genes showed differential expression in pathways involving cytokines. Significant associations between MFAP4 levels, immune infiltration, ferroptosis, and immune checkpoint genes were found in glioma tissues. MFAP4 levels were correlated with glioma stage, histological type, and 1p/19q status, and independently predicted overall survival (OS), disease-specific survival (DSS) and progression-free interval (PFI). MFAP4 expression is effective in distinguishing tumor tissue from normal brain tissue. Furthermore, Spearman Correlation emphasizes the significant relationship between MFAP4 and ferroptosis-related genes.

**Conclusion:**

Our study demonstrated that MFAP4 is aberrantly overexpressed in gliomas and correlates with adverse clinicopathological features. MFAP4 has relevance in regulating both tumor immunity and iron death, and cellular function assays have demonstrated that MFAP4 promotes the proliferation, migration, and invasion of glioma cells.

## 1 Introduction

Gliomas are a diverse group of tumors that originate from the glial cells of the central nervous system and are classified into several types and stages based on their histological features and molecular profiles (Chen et al., 2017). The World Health Organization (WHO) grading system categorizes gliomas into four grades (I-IV), with grade IV representing the most malignant form, glioblastoma multiforme (GBM) (Li et al., 2024). Other common types of gliomas include astrocytomas, oligodendrogliomas, and ependymomas, each with distinct pathological and genetic characteristics that influence their clinical behavior and response to therapy (Rezaei and Hanaei, 2023). The complex interactions between tumor cells and the immune microenvironment have attracted attention because it is well known that immune infiltration affects tumor progression and patient prognosis (Xu et al., 2020).

In recent years, immunotherapy has emerged as a novel cancer treatment strategy and has garnered widespread attention (Topalian et al., 2016). The core of immunotherapy lies in activating or enhancing the patient’s own immune system to recognize and attack cancer cells, thereby providing a more precise and potentially durable treatment option (Topalian et al., 2015). Compared with traditional surgery, chemotherapy, and radiotherapy, immunotherapy has significant advantages, including higher specificity, lower side effects, and the ability to elicit long-term immune memory (Fenis et al., 2024). However, despite the breakthrough progress achieved by immunotherapy in certain types of cancer, it still faces many challenges in the treatment of gliomas. Gliomas are highly invasive brain tumors with complex tumor microenvironments and immune-suppressive characteristics (Agosti et al., 2023). Glioma cells can escape recognition and attack by the immune system through various mechanisms, including the expression of immune-suppressive molecules, recruitment of immune-suppressive cells, and alteration of the composition of the extracellular matrix (Li et al., 2016). These characteristics limit the application of immunotherapy in gliomas but also provide new directions for research.

With the rapid advancement of molecular biology techniques, significant progress has been made in the study of molecular markers for gliomas (Bi et al., 2020). These molecular markers not only aid in the diagnosis and prognostic evaluation of gliomas but also provide a crucial basis for personalized treatment strategies (Reifenberger et al., 2017). Isocitrate dehydrogenase (IDH) mutations are among the most common molecular markers in gliomas, predominantly occurring in low-grade gliomas and secondary glioblastomas. IDH mutations hold substantial prognostic value, with patients harboring IDH-mutated gliomas typically exhibiting longer progression-free survival and overall survival (Rudà et al., 2024). The co-deletion of chromosomes 1p and 19q serves as a diagnostic molecular marker for oligodendrogliomas, with an incidence rate as high as 80%–90% in this tumor type. Glioma patients with 1p/19q co-deletions tend to respond well to radiotherapy and chemotherapy, resulting in relatively favorable prognoses (Eckel-Passow et al., 2015). In recent years, researchers have also identified several novel molecular markers, such as CDC6, PTPN1, HOTAIR, and GAS5 (Weller et al., 2024). These markers have demonstrated potential applications in the diagnosis and prognostic evaluation of gliomas. In summary, the detection and evaluation of these molecular markers are of great significance for the diagnosis, treatment selection, and prognostic assessment of gliomas (Gusyatiner and Hegi, 2018). With continuous technological advancements, it is anticipated that more molecular markers with clinical application value will be discovered, further advancing the precision treatment of gliomas. Notably, the identification of immune infiltration-associated genes associated with drug resistance remains an under-explored area, highlighting the need for further studies to elucidate the molecular pathways of immune evasion and drug resistance, which would pave the way for novel therapeutic strategies aimed at improving patient prognosis.

Microfibril associated protein 4 (MFAP4) is an extracellular matrix protein belonging to the family of fibrinogen-associated structural domains, which plays important biological roles in several organs, including the skeletal, vascular, hemostatic and immune systems (Pilecki et al., 2021). MFAP4 was originally thought to be a commonly missing gene in patients with Smith-Magenis syndrome (Zhao et al., 1995). Studies have shown that MFAP4 can be used for noninvasive assessment of a variety of diseases such as COPD(Johansson et al., 2014), liver cirrhosis (Mölleken et al., 2009), diabetes neuropathy (Blindbæk et al., 2017), cardiovascular complications (Wulf-Johansson et al., 2013). In addition, MFAP4 has been identified as an important regulator of lung adenocarcinoma (Wang et al., 2024) and pancreatic adenocarcinoma (Guerrero et al., 2021). However, its specific roles and mechanisms in gliomas are unknown and require further study. Detailed exploration of the functions and mechanisms of MFAP4 in gliomas is essential for gaining new insights and developing therapeutic strategies.

The main objective of this study is to comprehensively investigate the functions and mechanisms of MFAP4 associated with immunotherapy resistance in glioma. To achieve this goal, we will identify the specific functions of MFAP4 in glioma immunotherapy through multiple approaches. Specifically, our analyses will focus on the expression levels of MFAP4 in gliomas and its correlation with tumor immunotherapy responses. In addition, we will investigate the effects of MFAP4 on tumor immune evasion and drug resistance to reveal its mechanisms in gliomas. Our goal is to thoroughly elucidate the role of MFAP4 in immunotherapy resistance in glioma and provide substantial support for the development of more effective immunotherapeutic approaches.

## 2 Materials and methods

### 2.1 Datasets

RNA-seq data from the TCGA database (https://portal.gdc.cancer.gov) were downloaded and collated for the TCGA-GBM and TCGA-LGG Project STAR processes and extracted in TPM format. Differentially expressed genes were also evaluated in the GEO (http://www.ncbi.nlm.nih.gov/geo/) GSE7696 dataset in 70 patients with primary GBM and 10 patients with recurrent GBM after chemotherapy.

### 2.2 TIMER 2.0

The equations should be inserted in editable format from the equation editor. The TIMER 2.0 database allows the use of TCGA data to investigate differential expression of genes of interest between tumor and control tissues (Li et al., 2020). Thus, TIMER 2.0 was used to determine MFAP4 levels in various cancers.

### 2.3 TISIDB

The relationship between genomic expression levels and immune cell infiltration characteristics in tumors was analyzed using the TISIDB website (Ru et al., 2019). MFAP4 levels were analyzed in relation to various types of immune cells associated with tumors.

### 2.4 Protein-protein interaction analysis

STRING database is dedicated to predicting protein-protein interactions (PPI) and employs computational predictions, cross-organism knowledge transfer, and curated data from other databases to identify both direct and indirect associations (Szklarczyk et al., 2023). In this study, we utilized the STRING database to investigate protein-protein interactions.

### 2.5 Survival analysis

Kaplan-Meier plots, together with log-rank assessments, probed survival, utilizing the median level of MFAP4 expression as a cut-off value. The links across clinical features and survival were assessed through univariate/multivariate Cox regression with major parameters (*P* < 0.05) from the univariate analysis employed within the multivariate analysis. Forest maps were constructed using the R package “ggplot2.”

### 2.6 LASSO analysis

LASSO analysis was conducted as previously described (Ren et al., 2023). In this study, we utilized LASSO analysis to build a prognostic model and identify genes associated with prognosis.

### 2.7 Mutational analysis of MFAP4 in gliomas

The frequency of MFAP4 mutations in gliomas was assessed using cBioPortal, and specific types of mutations were analyzed using the COSMIC database.

### 2.8 Identification of differentially/co-expressed genes

Genes co-expressed with MFAP4 in gliomas were investigated using LinkedOmics (Vasaikar et al., 2018) and TCGA-derived RNA-seq datasets. Pearson’s correlation coefficient was used to explore co-expression, and volcano plots were generated from the LinkedOmics website. Heatmaps were created in R software (version 3.6.3) using limma. Patients in the TCGA dataset were assigned to the MFAP4 low and MFAP4 high groups based on median MFAP4 transcriptome levels, and differentially expressed genes (DEGs) were identified and plotted for all cohorts using the limma package. Venn diagrams were plotted to identify overlap between DEGs and co-expressed genes.

### 2.9 Functional enrichment analysis

The functions of the identified overlapping genes were explored by GO and KEGG analysis in MetScape (Zhou et al., 2019).

### 2.10 GSEA analysis

We employed the GSEA computational method to assess the statistical significance of a preselected gene set. Following correlation analysis, we generated an initial list of gene categories. These categories were then segmented into various groups for each analysis, involving, 1,000 permutations of gene sets to identify any disparities among them. The results of this analysis aided in identifying the critical genetic functions and signaling pathways linked to MFAP4.

### 2.11 Immune infiltration

Single-sample GSEA (ssGSEA) analysis was performed to detect the relationship between immune cell infiltration and gene expression in glioma tumors by means of the R-package GSVA package. Gene expression profiles of 24 immune cell types were studied. The association between MFAP4 levels and infiltrating cell subpopulations was tested by the Spearman and Wilcoxon rank sum test. The relationship between MFAP4 levels and immune cells was investigated by analyzing chemokines and their receptors using the “Chemokines” module in TISIDB.

### 2.12 Relationships between expression of MFAP4 and ferroptosis-associated genes

Potential relationships between the expression of MFAP4 and that of ferroptosis-associated genes were evaluated in TCGA-GBM/LGG datasets using R, which was also used to assess the proportions of ferroptosis-associated genes in samples with high or low MFAP4 levels. The “box plot” package in R was used to visualize the results.

### 2.13 Cell culture and experiments

The U251 and LN229 cell line was acquired from Servicebio Biotechnology Co. (Wuhan, China). The cells were maintained in DMEM with 10% fetal bovine serum and 10 μL/mL penicillin/streptomycin at 37°C and 5% CO_2_ with saturated humidity.

### 2.14 Transfection with small interfering RNA (siRNA)

U251 and LN229 cells (2 × 10^4^/well) were inoculated in 48-well plates and transfected with MFAP4 siRNA or siRNA negative control, as described in a manual (GenePharma, Shanghai, China). The siRNAs were mixed with 2.5 µL/100 µL of Lipofectamine 3,000 (Invitrogen, MA, United States) in a serum-free medium and allowed to stand for 20 min, after which the mixture was added to 250 µL medium containing 10% FBS. Lastly, 250 µL of this solution was placed in each well and incubated for 38 h under standard conditions. The MFAP4 siRNA sequences were for siMFAP4 #1: forward: 5′-CAC​UGA​AGC​AGA​AGU​AUG​ATT-3′ and reverse: 5′-UCA​UAC​UUC​UGC​UUC​AGU​GTT-3′, and #2: forward: 5′-CCA​CCU​CUC​UUA​UGC​CAA​UTT-3′ and reverse: 5′-AUU​GGC​AUA​AGA​GAG​GUG​GTT-3’. The control (si-Control) sequences were forward: 5′-UUC​UCC​GAA​CGU​GUC​ACG​UTT-3′ and reverse: 5′-ACG​UGA​CAC​GUU​CGG​AGA​ATT-3’. The siRNAs were synthesized by GenePharma Co., Ltd. (Shanghai, China).

### 2.15 The quantitative real-time polymerase chain reaction (qRT-PCR)

Total cellular RNA was extracted using Trizol reagent (Solarbio Biotechnology Co., Ltd., Beijing, China). Log-phase cells were taken. One milliliter of Trizol reagent was added to the cells, blown up with a pipette and ice-bath for 5 min to completely separate the nucleoprotein complexes. The content and purity of RNA were determined using a NanoDrop spectrophotometer (Thermo Fisher Scientific, United States). The purity of RNA was considered satisfactory when the OD_260 nm_/OD_280 nm_ values were between 1.8 and 2.0. The quantitative real-time polymerase chain reaction (qRT-PCR) was performed in two steps. The reaction conditions were as follows: pre-denaturation at 95.0°C for 30 s, denaturation at 95.0°C for 10 s, annealing and extension at 60.0°C for 30 s, and 40 cycles. The quantitative real-time PCR primers for MFAP4 and glyceraldehyde-3-phosphate dehydrogenase (GAPDH) were as follows: forward: 5′-GCT​GCT​GCT​GCT​TCT​CTC​CAC-3′, reverse: 5′-ACG​CCG​TCT​GAC​TGG​TAG​CC-3’; forward: 5′-CAG​GAG​GCA​TTG​CTG​ATG​AT-3′, reverse: 5′-GAA​GGC​TGG​GGC​TCA​TTT-3’. The relative expression of the target genes in each group was analyzed by the 2^−ΔΔCt^ method, where ΔΔCt = ΔCt (experimental group) - ΔCt (control group), ΔCt = Ct (target gene) - ΔCt (GAPDH), and Ct is the number of amplification cycles required for the fluorescence intensity to reach the threshold value.

### 2.16 Western blotting

U251 and LN229 cells were lysed in RIPA buffer (P0013B, Beyotime Biotechnology, Shanghai, China) containing PMSF (ST506, Beyotime Biotechnology) and phosphatase inhibitor (PPI, P1081, Beyotime Biotechnology). After separation on SDS-PAGE, the proteins were electroblotted onto PVDF membranes (0.45 µm, Merck Millipore Ltd., Darmstadt, Germany) and blocked for 1 h with 5% BSA. The blots were then treated with primary antibodies, rabbit anti-MFAP4 (17661-1-AP, Proteintech, Wuhan, China; 1:1,000) and rabbit anti-GAPDH (10494-1-AP, Proteintech, Wuhan, China; 1:10,000), followed by secondary antibodies. The bands were analyzed using a gel-imaging system (Tanon-4600, Tanon, Shanghai, China).

### 2.17 Cell proliferation and viability assay

Transfected U251 and LN229 cells (5,000/well) were grown in 96-well plates. After 24 h, 10 µL of CCK-8 solution (MCE, United States) was added, and absorbances at 450 nm were read after 2 h using a microplate reader (Thermo Fisher Scientific, Waltham, MA). Cell viability is represented by optical density (OD) values. Continuous testing for 5 days.

### 2.18 Colony formation assay

Different types of cells in the logarithmic growth phase counted, cells seeded at 500 pcs/well in 6-well plates, incubated with cells for 1 week. Observe the formation of punctate clones at the bottom of the 6-well plates, fix cells with 4% tissue fixative solution (Solarbio Biotechnology Co., Ltd., Beijing, China), stain 1% crystal violet for 30 min, and take pictures for analysis.

### 2.19 Wound-healing assays

Transfected U251 and LN229 cells were grown in 6-well plates until confluent. After removing detached cells by rinsing with PBS, scratches were made in the cell monolayer with a 200 µL pipette tip. The cells were kept in a serum-free medium and imaged after 0, 24, and 48 h. Data were analyzed using ImageJ 2.3.0.

### 2.20 Transwell assay

Suspend cells with serum-free medium, regulate the concentration of cells at 5 × 10^4^ pcs/well, seed in the upper chamber of the Transwell chamber, and spread 20% of the medium in the lower chamber. Incubate in 37°C 5%CO_2_ incubator for 24–48 h. Fix the upper chamber with 4% tissue fixative, stain with crystal violet for 30 min, wipe the inside of the upper chamber with a cotton swab, observe under a microscope and take pictures, count for analysis.

### 2.21 Statistical analysis

Bioinformatics data were assessed through R software (Version 3.6.3, R Foundation for Statistical Computing, Vienna, Austria). Differences were evaluated using *t*-tests and two-way ANOVA for separate specimens. *P* < 0.05 was deemed to confer statistical significance. Relationships between MFAP4 levels and patient clinicopathological features were analyzed by χ^2^ assessments, logistic regression, and Fisher’s exact and Wilcoxon rank-sum assessments.

## 3 Results

### 3.1 MFAP4 has been identified as an important marker associated with prognosis and treatment resistance

**FIGURE 1:**
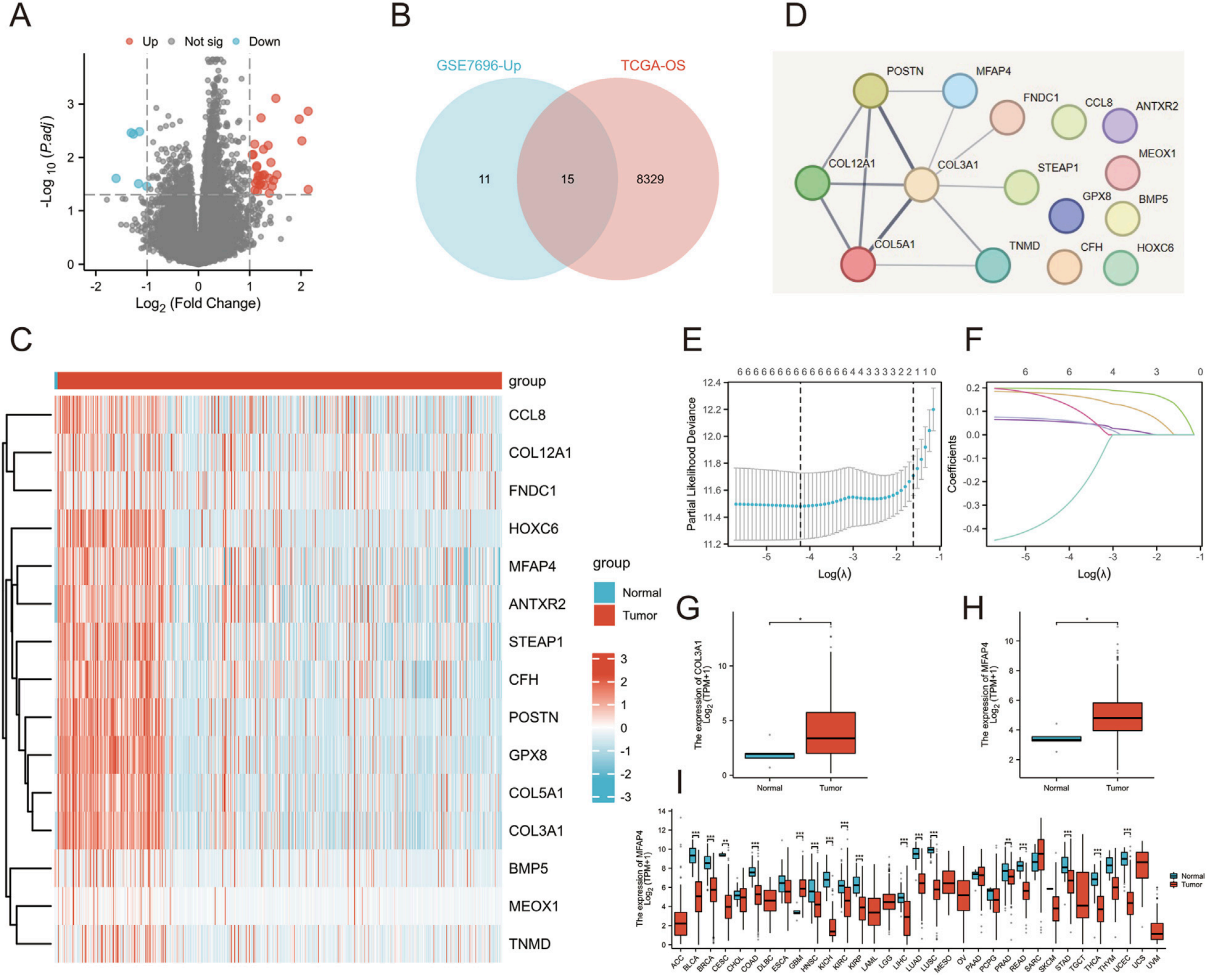
Integration of multiple datasets to identify markers associated with patient prognosis and treatment resistance. **(A)** Volcano plot revealing differential expression of genes in patients with primary GBM and patients with relapsed GBM after treatment in the GSE7696 dataset. **(B)** Venn diagram illustrating genes associated with overall survival and treatment resistance from two independent datasets. **(C)** Gene expression heatmap demonstrating the differential expression of genes in GBM patients and normal subjects. **(D)** PPI networks constructed by STRING revealed hub genes. **(E, F)** Results of Lasso regression analysis of prognosis-related genes. **(G)** Differential expression of COL3A1 in the TCGA-GBM/LGG database. **(H)** Differential expression of MFAP4 in the TCGA-GBM/LGG database. **(I)** Differential expression of MFAP4 in the TCGA pan-cancer database.

We downloaded the GSE7696 dataset from the GEO database, this dataset contained 70 patients with primary GBM and 10 patients with recurrent GBM after chemotherapy, which were processed to obtain significantly differentially expressed genes between the two groups of patients, of which 26 genes were significantly highly expressed in patients with recurrent GBM, and six were significantly lowly expressed in patients with recurrent GBM (Figure 1A). In addition, the information of GBM and LGG patient samples obtained from the TCGA database download was processed and analyzed to obtain the genes associated with the overall survival of patients, and a total of 8,345 genes encoding proteins were screened with *P* < 0.05 and HR > 1. Comparison of genes that were significantly highly expressed in recurrent GBM patients in GSE7696 yielded 15 genes co-existing in the two gene sets (Figure 1B). Heatmap difference analysis of these 15 genes was performed in the TCGA database (Figure 1C), while six key hub genes were obtained in the STRING database (Figure 1D): POSTN, MFAP4, COL3A1, TNMD, COL5A1, and COL12A1. Further examination using LASSO regression indicated that four key genes, including MFAP4, COL3A1, TNMD, and COL12A1, were significant (Figures 1E, F). Whether the expression differences were significant was further verified in the TCGA database (Normal,N = 55; Tumor,N = 701), and interestingly, only MFAP4 and COL3A1 were differentially expressed in normal and tumor patients (Figures 1G, H), and the remaining genes were not significantly differentially expressed (Supplementary Figure S1). COL3A1 has been shown to be associated with poor prognosis and drug resistance in GBM patients (Gao et al., 2016), so we mainly focused on whether MFAP4 plays an important role in the development of gliomas. In addition, MFAP4 was significantly differentially expressed in a variety of other cancers including colorectal and gastric cancers (Figure 1I). It is important to note that when there are sample sizes of less than 3 or a standard deviation (SD) of 0 within a group within a data subgroup (SARC, SKCM, THYM, ACC, DLBC, LAML, LGG, MESO, OV … ), these groups will not be included for statistical analysis. We also analyzed the dependence of cell lines on MFAP4 in DepMap, and the results suggest that MFAP4 has an important role in brain tumor types and may be a potential therapeutic target (Supplementary Figure S2). Overall, MFAP4 as a prognostic marker was positively associated with treatment resistance.

### 3.2 MFAP4 is aberrantly overexpressed in gliomas and correlates with adverse clinicopathologic features of gliomas

**FIGURE 2:**
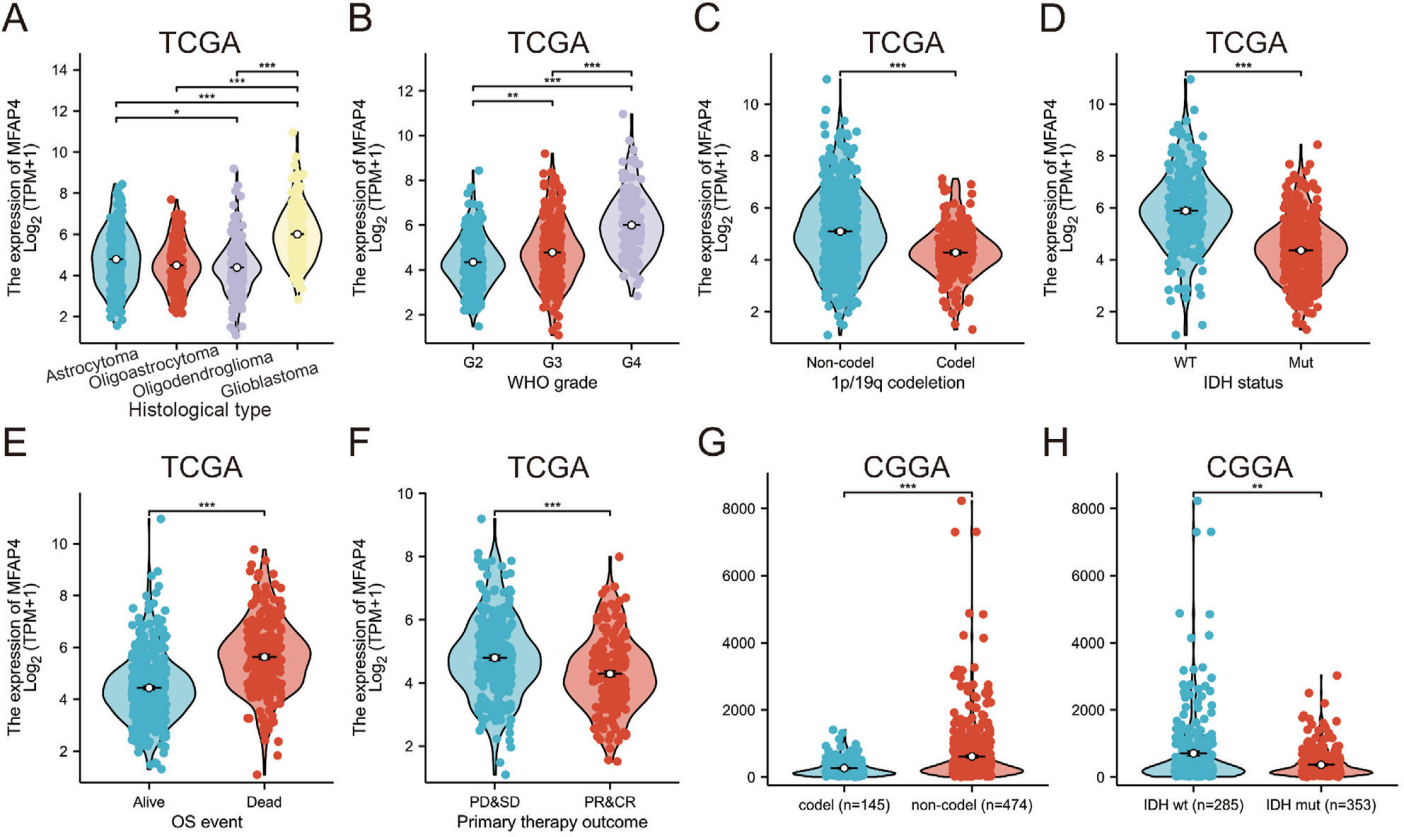
The Cancer Genome Atlas (TCGA) and Chinese Glioma Genome Atlas (CGGA) databases reveal that MFAP4 is aberrantly overexpressed in gliomas **(A–F)** MFAP4 expression in TCGA glioma patients with different histological types **(A)**, WHO grade **(B)**, 1p/19q codeletion **(C)**, isocitrate dehydrogenase (IDH) status **(D)**, overall survival events **(E)**, and primary therapy outcome **(F)**. **(G, H)** Expression of MFAP4 in CGGA in different 1p/19q codeletion **(G)** and in different IDH genotypes **(H)** were analyzed.

Clinical information and MFAP4 levels of GBM/LGG patients were obtained by TCGA. Associations between these parameters were examined by univariate analysis and significant associations were found between elevated MFAP4 levels and WHO classification, IDH genotype, 1p/19q codeletion, primary therapy outcome and age (Table 1). Gene expression profiles of all types of tumor samples showed that MFAP4 expression was significantly elevated in high-grade gliomas, especially in GBM (Figure 2A), and furthermore, MFAP4 expression increased with WHO grade (Figure 2B). Molecular markers, such as 1p/19q codeletion and IDH genotypes, have been widely used for diagnosis and prognostic assessment of gliomas (Cheng et al., 2023). We found that messenger RNA (mRNA) levels of MFAP4 were enriched in 1p/19q non-codel (Figure 2C) and IDH wild-type (Figure 2D) cases. Importantly, overexpression of MFAP4 in the TCGA dataset was significantly associated with WHO classification, IDH genotype, 1p/19q codeletion, primary therapy outcome, age, and histologic type (Table 2). In OS events, MFAP4 mRNA expression was higher in the death group than in the live group (Figure 2E), and in treatment outcome, MFAP4 mRNA expression was higher in the PD&SD group than in the PR&CR group (Figure 2F). In particular, MFAP4 expression was validated in the CGGA dataset for the 1p/19q codeletion and IDH genotype (Figures 2G, H).

**TABLE 1:** Links across MFAP4 levels and Glioma clinicopathological characteristics, shown through logistic regression assessment.

Characteristics	Total (N)	OR (95% CI)	P value
WHO grade (G3&G4 vs. G2)	637	3.478 (2.465–4.908)	<0.001
IDH status (Mut vs. WT)	689	0.125 (0.086–0.181)	<0.001
1p/19q codeletion (Codel vs. Non-codel)	692	0.272 (0.186–0.398)	<0.001
Primary therapy outcome (PR&CR vs. PD&SD)	465	0.464 (0.317–0.680)	<0.001
Gender (Male vs. Female)	699	1.005 (0.745–1.356)	0.974
Race (Black or African American and White vs. Asian)	686	1.595 (0.517–4.926)	0.417
Age (>60 vs. ≤ 60)	699	3.221 (2.156–4.814)	<0.001

**TABLE 2:** Association between MFAP4 mRNA expression and the clinical parameters of glioma patients from The Cancer Genome Atlas (TCGA).

Characteristics	Low expression of MFAP4 n (%)	High expression of MFAP4 n (%)	P value
WHO grade (N = 539)			<0.001
G2	153 (24%)	71 (11.1%)	
G3	126 (19.8%)	119 (18.7%)	
G4	32 (5%)	136 (21.4%)	
IDH status (N = 689)			<0.001
WT	49 (7.1%)	197 (28.6%)	
Mut	295 (42.8%)	148 (21.5%)	
1p/19q codeletion (N = 692)			<0.001
Non-codel	222 (32.1%)	298 (43.1%)	
Codel	126 (18.2%)	46 (6.6%)	
Primary therapy outcome (N = 465)			<0.001
PD&SD	130 (28%)	130 (28%)	
PR&CR	140 (30.1%)	65 (14%)	
Gender (N = 699)			0.974
Female	149 (21.3%)	149 (21.3%)	
Male	200 (28.6%)	201 (28.8%)	
Race (N = 686)			0.413
Asian	8 (1.2%)	5 (0.7%)	
Black or African American and White	337 (49.1%)	336 (49%)	
Age (N = 699)			<0.001
≤60	309 (44.2%)	247 (35.3%)	
>60	40 (5.7%)	103 (14.7%)	
Histological type (N = 699)			<0.001
Astrocytoma	96 (13.7%)	100 (14.3%)	
Oligoastrocytoma	84 (12%)	51 (7.3%)	
Oligodendroglioma	137 (19.6%)	63 (9%)	
Glioblastoma	32 (4.6%)	136 (19.5%)	

### 3.3 MFAP4 is an independent prognostic indicator significantly associated with disease progression in gliomas

**FIGURE 3:**
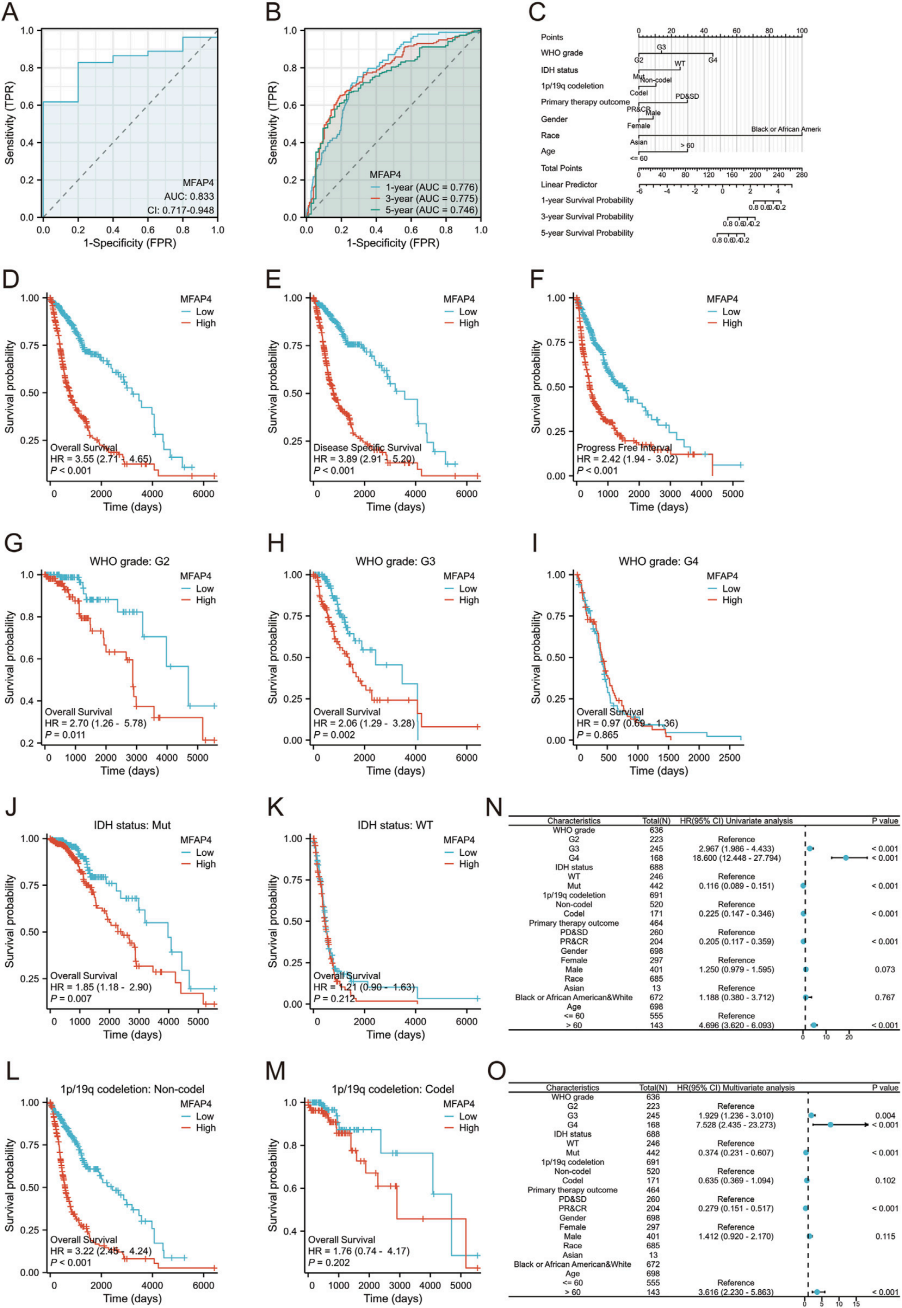
MFAP4 is a negative predictor of survival in glioma patients. **(A)** ROC curves of MFAP4 expression between tumor tissues and control tissues. **(B)** Time-dependent ROC curves for predicting 1-, 3-, and 5-year survival based on MFAP4 levels. **(C)** A nomogram including various clinicopathologic parameters (WHO grade, IDH status, 1p/19q codeletion, primary therapy outcome, and age) and MFAP4 expression levels predicted efficacy for 1-, 3-, and 5-year survival outcomes. **(D–F)** Overall survival analysis **(D)**, disease-specific survival (DSS) analysis **(E)** and progression-free interval (PFI) analysis **(F)** of the TCGA dataset **(G–I)** Survival analysis of MFAP4 mRNA expression in patients with different WHO grade in the TCGA dataset. **(J, K)** Survival analysis of MFAP4 mRNA expression in different IDH status in the TCGA dataset **(L, M)** Survival analysis of MFAP4 mRNA expression in different 1p/19q codeletion of the TCGA dataset. **(N, O)** Forest plots showing univariate **(N)** and multivariate **(O)** Cox regression models for MFAP4 overall survival in the TCGA cohort.

Analysis of the ROC curve for MFAP4 showed an area under the ROC curve (AUC) of 0.833, indicating that MFAP4 was able to accurately differentiate between tumor and normal control samples, and that its expression in gliomas has a high diagnostic value (Figure 3A). Time correlation analysis of the ROC curve showed that MFAP4 predicted 1-, 3-, and 5-year survival with an AUC value greater than 0.7 (Figure 3B). Then, we constructed a nomogram based on the significant correlation of MFAP4 level, WHO grade, IDH genotype, 1p/19q codeletion, primary therapy outcome, and age, and determined that these metrics had a significant correlation with patients’ prognosis by multifactorial analysis, and we found that the nomogram was effective in predicting 1-, 3-, and 5-year survival rates of glioma patients (Figure 3C). OS analysis of the TCGA dataset showed that MFAP4 was negatively correlated with the prognosis of glioma (Figure 3D). In the TCGA dataset, MFAP4 expression was also significantly associated with DSS and PFI in glioma patients (Figures 3E, F). In addition, patients with elevated MFAP4 expression in the WHO G2, G3, IDH mutation groups and 1p/19q non-codel group had a poorer prognosis (Figures 3G–M). One-way Cox regression analysis showed that WHO grade, IDH status, 1p/19q codeletion, primary therapy outcome and age were associated with OS in glioma patients (Figure 3N). Multifactorial Cox regression analysis showed that WHO grade, IDH status, primary therapy outcome and age were associated with OS in glioma patients (Figure 3O). Univariate and multifactorial Cox regression analyses of DSS and PFI in glioma patients are shown in Supplementary Figure S3. These results strongly suggest that MFAP4 is an independent prognostic factor associated with disease progression in glioma.

### 3.4 Mutations in MFAP4 are not associated with survival

**FIGURE 4:**
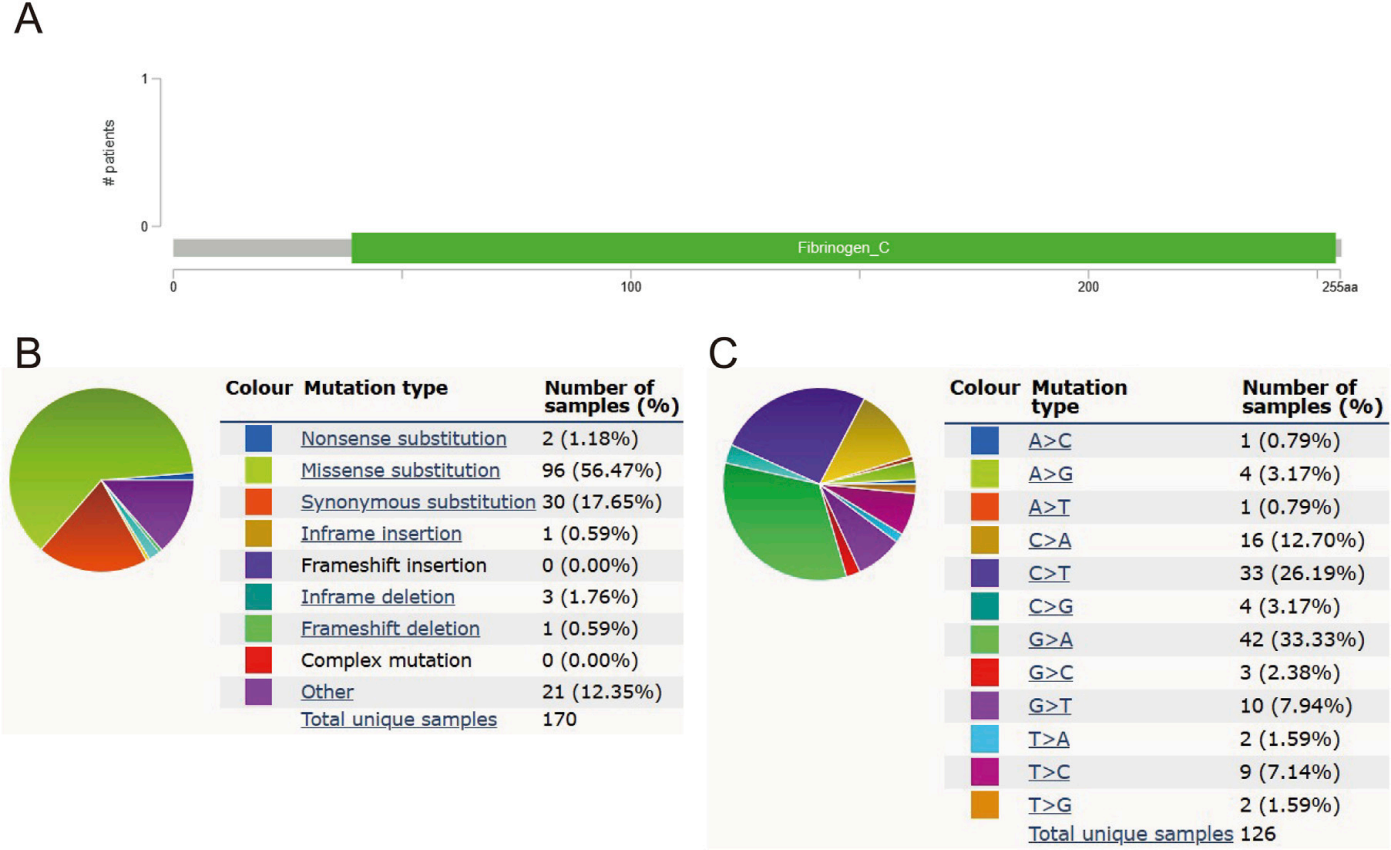
MFAP4 mutations in gliomas. **(A)** Schematic representation of MFAP4 mutations in gliomas, as determined by cBioPortal. **(B)** MFAP4 mutation types identified using the COSMIC database. **(C)** Frequency of MFAP4 mutations identified by the COSMIC database.

MFAP4 mutation frequency was assessed using the cBioPortal online resource. 1,309 samples from 1,229 glioma patients in the MSK, CPTAC, and TCGA datasets were analyzed. The overall frequency of MFAP4 somatic mutations associated with gliomas was 0, suggesting no association between MFAP4 mutations and prognosis of glioma patients (Figure 4A). We also evaluated the types of MFAP4 mutations using the COSMIC database, and approximately 56.47% of the samples contained missense mutations and 17.65% contained synonymous mutations (Figure 4B). The majority of substitutions were G>A (33.33%) (Figure 4C).

### 3.5 Functional annotation and pathway enrichment of MFAP4-related genes in gliomas

**FIGURE 5:**
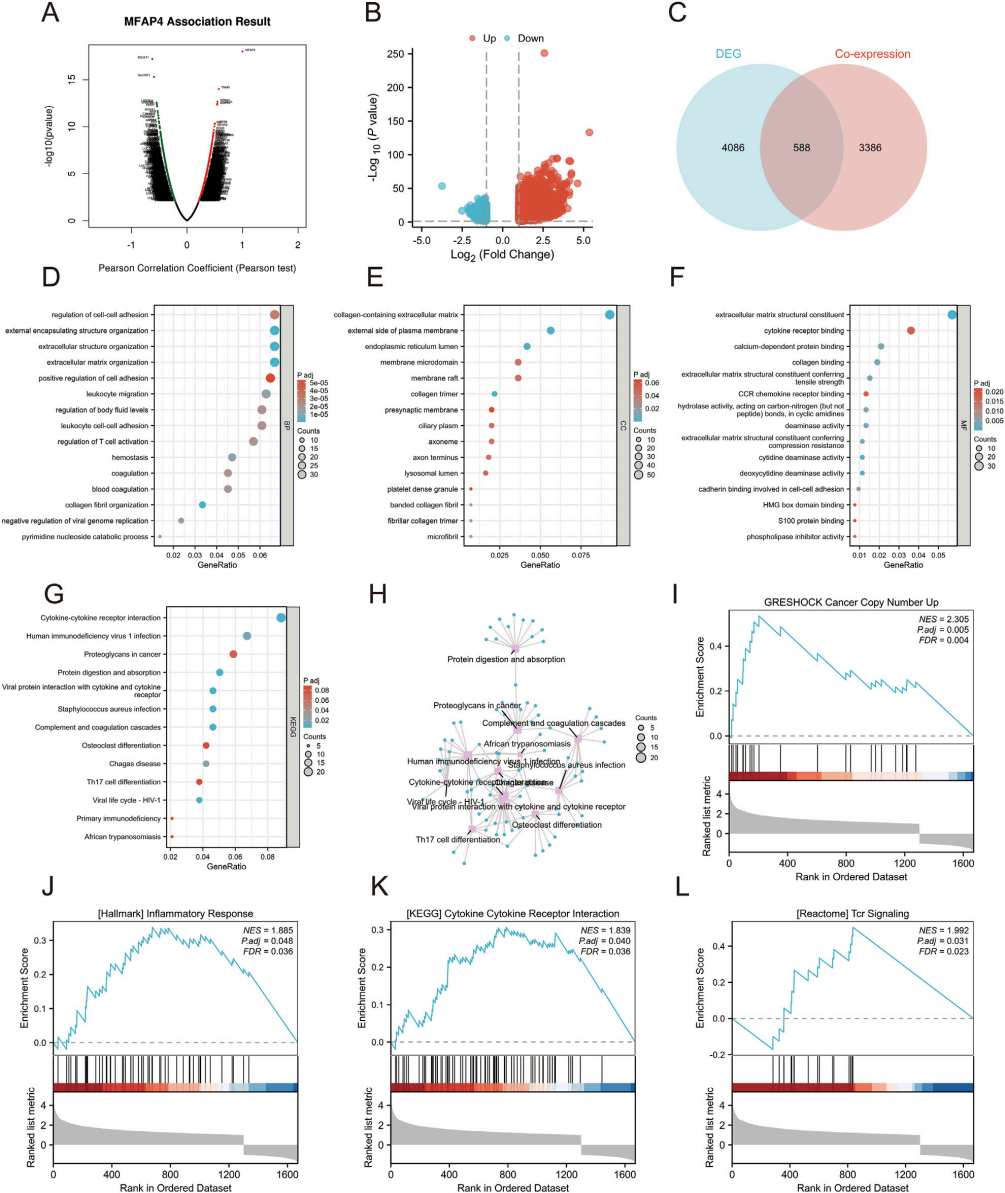
Gene set enrichment analysis (GSEA) of MFAP4 in the Gene Ontology (GO), Kyoto Encyclopedia of Genes and Genomes (KEGG), and TCGA datasets. **(A)** Volcano plot showing genes co-expressed with MFAP4 found in TCGA. **(B)** Volcano plot showing DEGs identified between the MFAP4 High and MFAP4 Low groups in TCGA-GBM/LGG. **(C)** Venn diagram reveals overlapping genes between the two groups. **(D–F)** GO enrichment analysis of biological processes **(D)**, cellular components **(E)** and molecular functions **(F)** of MFAP4 and its co-expressed genes. **(G, H)** KEGG pathway enrichment analysis of MFAP4 and its co-expressed genes **(I–L)** GSEA enrichment analysis of MFAP4 and its co-expressed genes.

To explore the possible function of MFAP4 in gliomas, we further explored the genes found to be co-expressed with MFAP4 in TCGA using LinkedOmics (Figure 5A). This showed a significant correlation between MFAP4 and the 3,974 genes co-expressed in gliomas (FDR<0.05, *P* < 0.05). The DEGs identified between the MFAP4 High and MFAP4 Low groups in gliomas were then examined. A total of 4,674 DEGs were observed in the MFAP4 High group (*P* < 0.05, |log_2_ FC|≥1) (Figure 5B), of which 3,785 DEGs were upregulated and 889 DEGs were downregulated. A Venn diagram was used to compare DEGs with the overlap found between genes co-expressed with MFAP4, which showed 588 genes overlapped between the two populations (Figure 5C). Next, we functionally analyzed these genes to elucidate the possible role of MFAP4. The results showed that MFAP4 co-expressed genes were mainly enriched in immune-related biological processes such as regulation of cell adhesion, positive regulation of cell adhesion, leukocyte migration, leukocyte-cell adhesion, and activation of T cells (Figure 5D). Among the cellular components, collagen-containing extracellular matrix, endoplasmic reticulum lumen, and presynaptic membrane were enriched (Figure 5E). MFAP4 co-expressed genes were mainly enriched for immune-related molecular functions, such as cytokine receptor, chemokine receptor binding, etc. (Figure 5F). The enrichment analysis of KEGG pathway showed that MFAP4 co-expressed genes were involved in cytokine-receptor interactions, tumor proteoglycans, viral proteins with cytokines and receptor interactions, complement and cohesion cascades, and Th17 cell differentiation pathways were highly enriched (Figures 5G, H). GSEA also showed significant enhancement of cancer copy number (the top 5 enriched gene sets: SP1, CHD1, KAT6, EP300, CREBBP), inflammatory response (the top 5 enriched gene sets: IL1B, CXCL8, COX2, NFKB1, STAT1), cytokine receptor interactions (the top 5 enriched gene sets: IL2, IFNG, CSF2, IL2RA, IFNGR1), and TCR signaling pathways (the top 5 enriched gene sets:ZAP70, LCK, LAT, PIK3R1, AKT1) (Figures 5I–L). These results suggest that MFAP4 may be involved in the tumor immune microenvironment and immune regulation.

### 3.6 MFAP4 levels and immune cell infiltration

**FIGURE 6:**
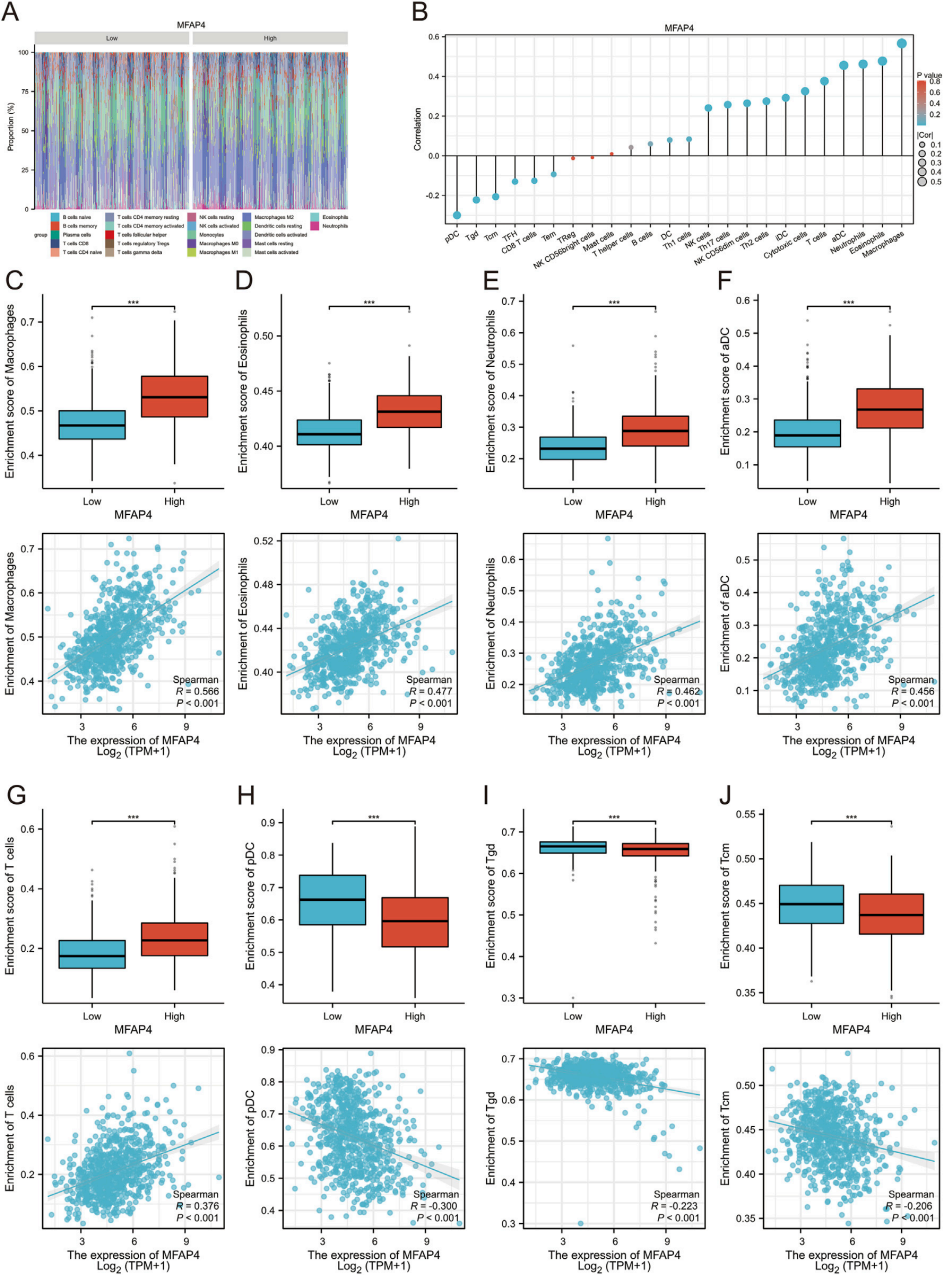
Relationship between MFAP4 levels and immune cell infiltration in gliomas. **(A, B)** Correlation of different types of immune cell infiltration with MFAP4 levels using the CIBERSORT method **(A)** and ssGsea method **(B, C–J)** The degree of infiltration of macrophages **(C)**, eosinophils **(D)**, neutrophils **(E)**, activated dendritic cells **(F)**, T cells **(G)**, pDC cells **(H)**, Tgd cells **(I)**, and Tcm cells **(J)** in the high/low MFAP4 cohort.

To further validate the correlation between MFAP4 levels and immune cell infiltration, we used CIBERSORT to detect the difference in immune cell infiltration in groups with different MFAP4 expression levels, and plotted an immune infiltration superimposed histogram (Figure 6A). Glioma infiltration of 24 immune cell types was detected using ssGSEA and its correlation with MFAP4 levels was assessed by Spearman’s correlation coefficient. MFAP4 was positively correlated with macrophages (R = 0.566, *P* < 0.001), eosinophils (R = 0.477, *P* < 0.001), neutrophils (R = 0.462, *P* < 0.001), activated dendritic cells (R = 0.456, *P* < 0.001), and T cells (R = 0.376, *P* < 0.001). In contrast, pDC cells (R = −0.3, *P* < 0.001), Tgd cells (R = −0.223, *P* < 0.001), and Tcm cells (R = −0.206, *P* < 0.001) were negatively correlated with MFAP4 (Figure 6B). Infiltration of macrophages (Figure 6C), eosinophils (Figure 6D), neutrophils (Figure 6E), activated dendritic cells (Figure 6F), T cells (Figure 6G), pDC cells (Figure 6H), Tgd cells (Figure 6I), and Tcm cells (Figure 6J) was in agreement with the correlation of Figure 6B.

### 3.7 MFAP4 expression in relation to immunoinhibtors, chemokines and chemokine receptors

**FIGURE 7:**
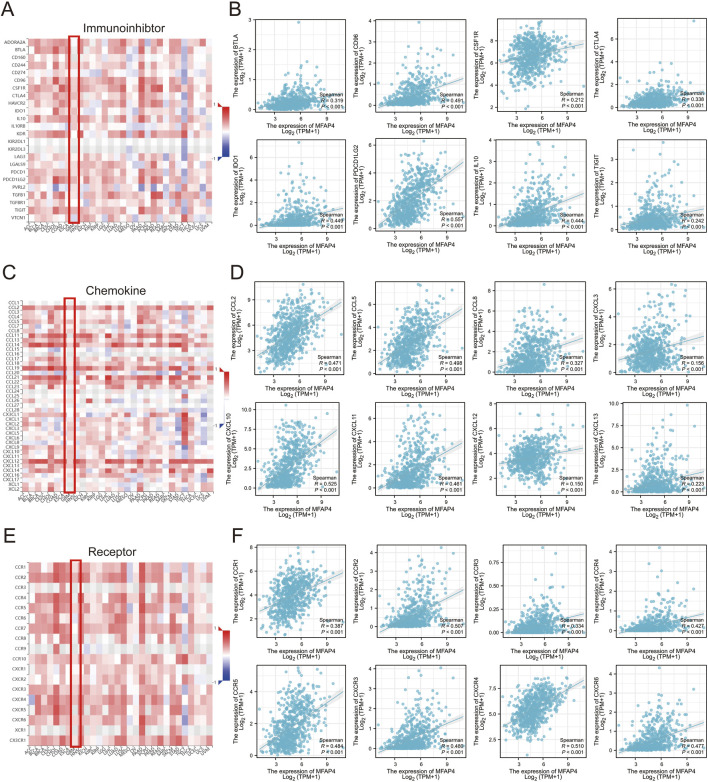
Correlation of MFAP4 expression with immunoinhibitors, chemokines, and chemokine receptors expression. **(A, C, E)** Correlation of MFAP4 with immunoinhibtors **(A)**, chemokines **(C)**, and chemokine receptors **(E)** expression in human cancers. **(B, D, F)** Correlation of MFAP4 with immunoinhibitors **(B)**, chemokines **(D)**, and chemokine receptors **(F)** expression in gliomas from The Cancer Genome Atlas (TCGA) dataset.

To further explore the role of MFAP4 in tumor immune response, we used TISIDB to calculated the correlation of MFAP4 expression with immunoinhibtors, chemokines and chemokine receptors. The results showed that MFAP4 was positively correlated with a variety of immunoinhibtors (Figure 7A), such as BTLA (R = 0.319, *P* < 0.001), CD96 (R = 0.491, *P* < 0.001), CSFIR (R = 0.212, *P* < 0.001), CTLA4 (R = 0.338, *P* < 0.001), IDO1 (R = 0.449, *P* < 0.001), IL10 (R = 0.557, *P* < 0.001), PDCD1LG2 (R = 0.444, *P* < 0.001), TIGIT (R = 0.242, *P* < 0.001). We also analyzed the correlation between MFAP4 expression and chemokines and their receptors. The expression of MFAP4 was positively correlated with the major chemokines, including CCL2, CCL5, CCL8, CCL14, CCL19, CCL22, CXCL2, CXCL3, CXCL6, CXCL9, CXCL10, CXCL11, CXCL12, CXCL13, etc (Figure 7C). In addition, MFAP4 expression was also positively correlated with major chemokine receptors such as CCR1, CCR2, CCR3, CCR4, CCR5, CXCR3, CXCR4, and CXCR6 (Figure 7E). In addition, we correlated MFAP4 with the expression of immunoinhibtors (Figure 7B), chemokines (Figure 7D), and chemokine receptors (Figure 7F) in gliomas from The Cancer Genome Atlas (TCGA) dataset. Our findings suggest that MFAP4 may play an important role in tumor immunomodulation and may serve as a drug target for glioma.

### 3.8 Study of the correlation between MFAP4 and ferroptosis in gliomas

**FIGURE 8:**
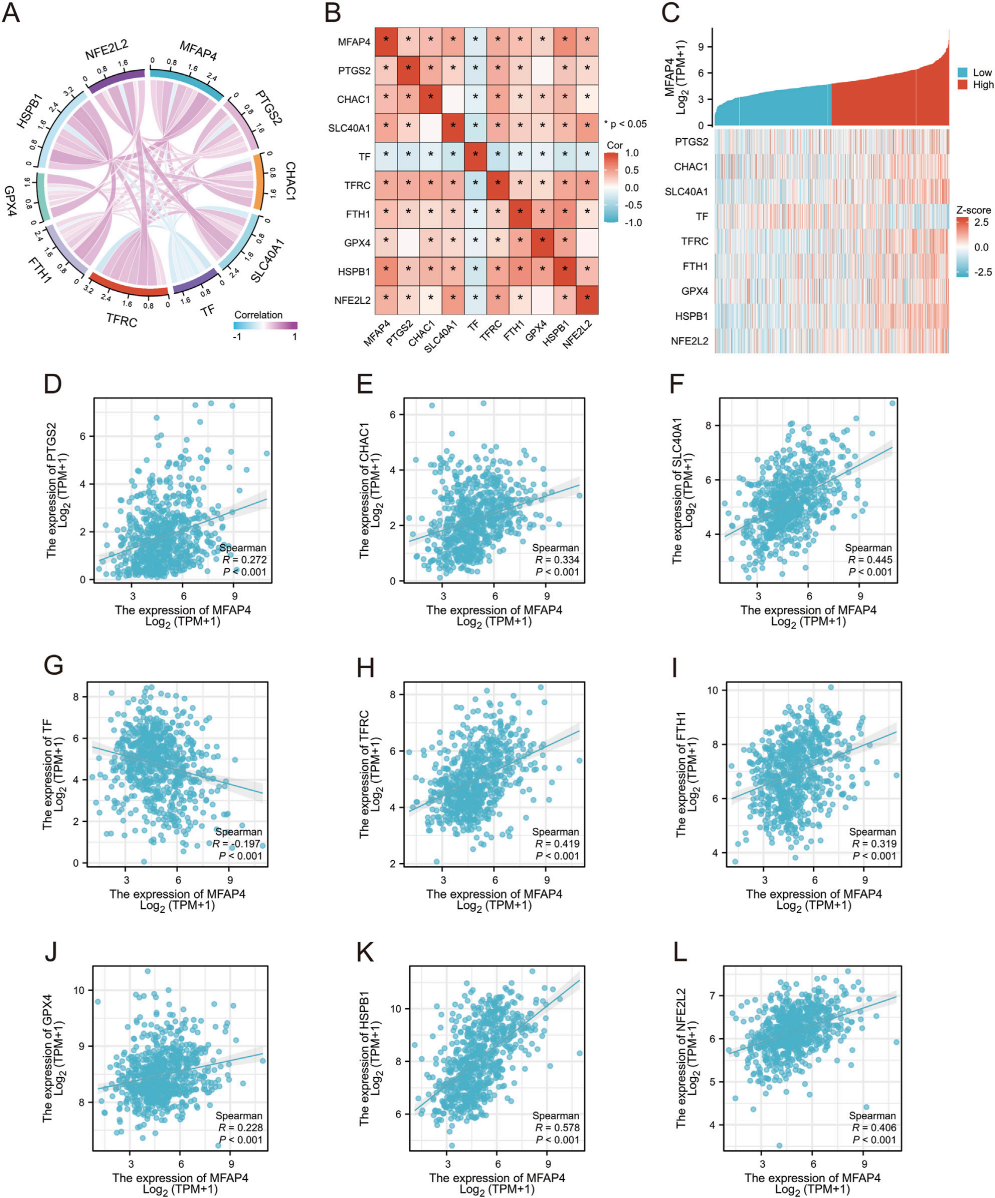
Relationship between MFAP4 and expression of ferroptosis -related genes in gliomas. **(A, B)** TCGA data showing the correlation of MFAP4 with ferroptosis markers. **(C)** Correlation of MFAP4 with ferroptosis markers using GEPIA2 data. **(D–L)** Differential expression of ferroptosis markers in glioma samples in the high MFAP4 and low MFAP4 groups.

Ferroptosis was first described in 2012 (Dixon et al., 2012), and unlike other death-related processes such as apoptosis and autophagy, it is iron-dependent and promoted by reactive oxygen species (ROS). It is associated with a variety of cellular changes, particularly in mitochondria, including cristae reduction, disruption of the outer mitochondrial membrane, and membrane compaction (Mou et al., 2019). Ferroptosis is regulated by multiple genes and pathways associated with cancer, suggesting that induction of ferroptosis may be a potential strategy to halt cancer progression. Importantly, ferroptosis is also involved in immunotherapy resistance in cancer (Yin et al., 2023). Thus, we used the TCGA-GBM/LGG dataset to assess the association between MFAP4 levels and ferroptosis, and showed significant associations between MFAP4 levels and ferroptosis markers including PTGS2, CHAC1, SLC40A1, TF, TFRC, GPX4, NFE2L2, HSPB1, and FTH1 (Figures 8A, B). TCGA-GBM/LGG samples were categorized into low and high MFAP4 expression groups, and differentially expressed ferroptosis-related genes were identified (Figure 8C). The results revealed that the expression of PTGS2, CHAC1, SLC40A1, TFRC, FTH1, GPX4, HSPB1, and NFE2L2 was upregulated in the high MFAP4 group (*P* < 0.05), and the TF expression level was reduced (*P* < 0.05) (Figures 8D–L). Thus, MFAP4 may influence glioma progression and prognosis through its ability to regulate ferroptosis.

### 3.9 MFAP4 regulates proliferation, migration and invasion of gliomas cells

**FIGURE 9:**
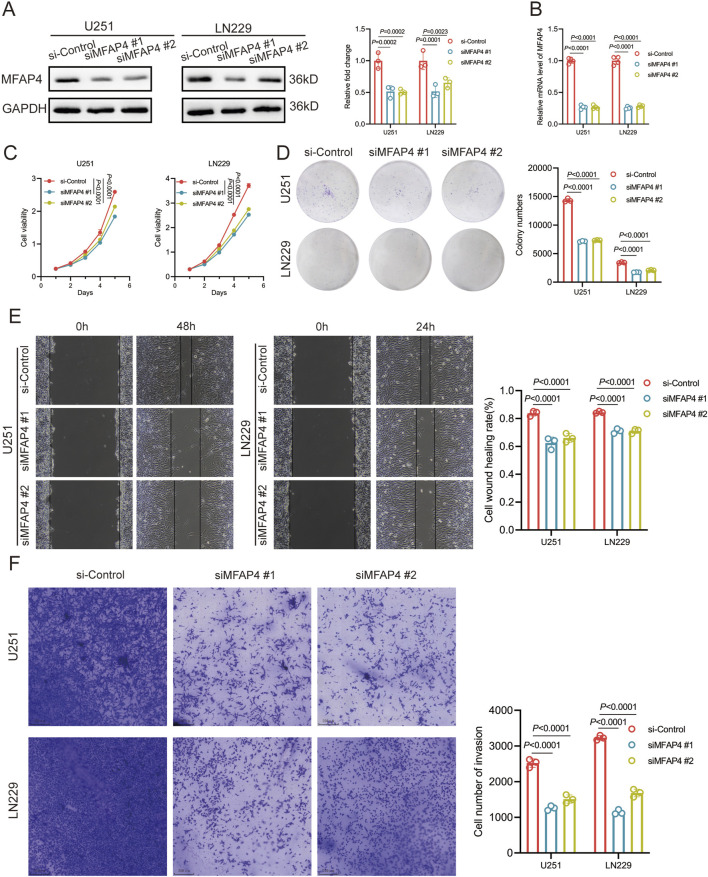
MFAP4 regulates proliferation, migration and invasion of GBM cells. **(A, B)** Down expressed MFAP4 in U251 and LN229 cells was confirmed by Western blotting **(A)** and qRT-PCR **(B)**. **(C)** CCK-8 assay was used to detect the proliferation of U251 and LN229 cells. **(D)** Colony formation assay was used to test the proliferative capacity of U251 and LN229 cells. **(E)** Wound-healing assay was used to test the migratory capacity of U251 and LN229 cells. **(F)** Transwell assay used to detect the invasive ability of U251 and LN229 cells.

To further evaluate the role of MFAP4, we knocked down MFAP4 in U251 and LN229 cells and verified the knockdown efficiency by Western blotting and qRT-PCR (Figures 9A, B). CCK-8 assay showed that the cell viability was significantly reduced after MFAP4 knockdown (Figure 9C). The results of colony formation assay showed that the proliferation ability of cells was significantly reduced after knockdown of MFAP4 (Figure 9D). Cell migration was detected by wound healing assay, and it was found that the migration ability of U251 and LN229 cells was significantly reduced after knockdown of MFAP4 (Figure 9E). In addition, cell invasion was detected by Transwell assay, and it was found that the invasion ability of U251 and LN229 cells was significantly reduced after knockdown of MFAP4 (Figure 9F). These results indicated that MFAP4 was involved in cell proliferation, migration and invasion of gliomas cells.

## 4 Discussion

In recent years, the rapid development of bioinformatics has greatly improved the diagnosis and prognosis of diseases (Chi et al., 2023). In this study, MFAP4 was identified as a potential biomarker for prognosis and immunotherapy in glioma patients by bioinformatics analysis. Microfibril associated protein 4 (MFAP4) is an extracellular matrix protein belonging to the fibrinogen-associated structural domain family, which includes a variety of proteins involved in tissue homeostasis and innate immunity (Han Y. et al., 2021). In addition, MFAP4 specifically binds ECM protofibrils, protofibrillar proteins, elastin, and collagen (Pilecki et al., 2016), and it activates a variety of cells through RGD-dependent integrin ligation and downstream focal adhesion kinase (FAK)-dependent signaling (Pilecki et al., 2015). Although it has been implicated in the progression and development of certain cancers, the exact biological function and role of MFAP4 in disease pathogenesis remains incompletely understood, suggesting the need for further studies to identify it as a potential therapeutic target. In the context of gliomas, few previous studies have focused on the role of MFAP4 despite its expression in many human cancers. To address this gap, we analyzed transcriptomic data from different databases such as TCGA, GTEx and GEO to explore the potential impact of MFAP4 in gliomas. Interestingly, MFAP4 is highly expressed only in gliomas and diametrically opposite in other cancers, which may be due to the fact that gliomas are highly heterogeneous tumors whose development involves a variety of molecular mechanisms. MFAP4 may play a key role in specific pathological processes in gliomas or may have a tissue-specific expression pattern, which does not have a similar function in other cancers (Huang et al., 2010). Or perhaps the tumor microenvironment (TME) of gliomas is significantly different from other cancer types. In addition, differences in the mechanisms regulating gene expression may contribute to this phenomenon. In gliomas, certain genes may be subject to specific epigenetic regulation. For example, mutations in the IDH gene, which are more common in gliomas, cause alterations in DNA methylation levels that affect gene expression (Wu et al., 2024). This epigenetic regulatory mechanism may activate certain genes specifically in gliomas, whereas a similar pattern of regulation does not exist in other cancers. However, the specific activation mechanism of MFAP4 in gliomas needs to be explored and investigated in deeper experimental areas.

Poor prognosis and tumor recurrence in cancer patients are modulated by a variety of factors, including alterations in the ability of tumor cells to migrate (Li et al., 2023). Therefore, it is crucial to identify these potential regulators. With this in mind, the present study conducted a comprehensive bioinformatics-based analysis to explore their possible functional and diagnostic roles in this context. In this study, significant correlations were found between MFAP4 levels in tumor samples and WHO grade, IDH status, 1p/19q codeletion, primary therapy outcome, age and survival. In addition, high levels of MFAP4 expression were associated with poorer patient prognosis and later clinical grading of the tumor. Logistic regression analysis showed significant associations between MFAP4 levels and WHO grade, IDH status, 1p/19q codeletion, primary therapy outcome, and age. Kaplan-Meier curves showed that patients with higher MFAP4 levels had significantly lower OS, DSS, and PFI. The association between MFAP4 and poor prognosis has been observed in a variety of cancer types, including prostate cancer, limited breast cancer, and bladder cancer (Li et al., 2022). The results of such a dataset suggest that MFAP4 may be a prognostic biomarker for many cancers, including gliomas.

The immune system plays a crucial role in cancer development (Xiao et al., 2020). To further understand the effect of MFAP4 on TME, we further investigated the possible role of MFAP4 in gliomas using GSEA and GO analyses, which showed that it is involved in multiple immune-related pathways including leukocyte migration, T cell activation, cytokine receptor interaction and chemokine receptor binding, which suggests that MFAP4 may regulate the tumor micro environment affecting the level of immune infiltration and thus drug resistance in glioma patients. The importance of genetic and epigenetic inheritance on cancer development is well known (Deng et al., 2023). However, we found an overall frequency of 0 for MFAP4 somatic mutations associated with gliomas, suggesting that there is no association between MFAP4 mutations and the prognosis of glioma patients.

The tumor microenvironment consists not only of tumor cells, but also a variety of immune and stromal cells, including fibroblasts, endothelial cells, and neurons (Hinshaw and Shevde, 2019). The type/degree of immune cell infiltration can help predict a patient’s response to immunotherapy. Here, we determined an association between immune cell infiltration and MFAP4 levels, observing that MFAP4 was negatively correlated with the number of toxic cells such as pDC cells, Tcm cells, and CD8^+^ T lymphocytes. These toxic cells are capable of activating immune responses against tumors, leading to tumor cell lysis (Banerjee et al., 2021). It has been reported that pDC are able to regulate tumorigenesis through the action of the chemokine receptor CXCR4 (Han N. et al., 2021). In addition, Tcm cell survival, proliferation and differentiation require cytokine regulation. By responding to signals from these cytokines, Tcm cells are able to perform their key function as memory cells, providing long-term immune protection to the body (Klebanoff et al., 2016). MFAP4 levels also correlate with the concentrations of many chemokines and chemokine receptors as well as immune checkpoints in glioma tissues, suggesting that it may influence the tumor microenvironment through multiple mechanisms composition. Thus, our findings suggest that elevated MFAP4 levels are closely associated with mechanisms that promote immune escape in glioma tumor cells, thereby promoting tumor growth and progression.

Ferroptosis has been suggested as a target for the treatment of cancer, especially for refractory tumors (Dixon et al., 2012),and as a mechanism for resistance to cancer immunotherapy (Xiang et al., 2024). Many tumor suppressor proteins, such as P53, fumarase, and BAP1, sensitize tumor cells to ferroptosis (Mou et al., 2019). In this paper, we investigated the relationship between MFAP4 levels and ferroptosis-sensitization-related genes and found a positive correlation. These results suggest that MFAP4 promotion of tumorigenesis is related to the ability of MFAP4 to regulate ferroptosis, which may provide a new direction for targeting iron prolapse in the treatment of glioma.

These data suggest that MFAP4 may serve as a biomarker with potential as an integrative or specific target for glioma cancer immunotherapy. From the correlation analysis of MFAP4 with immune infiltration, immune checkpoint inhibitors, chemokines, and chemokine receptor expression in gliomas, it is clear that MFAP4 may play an important role in tumor immunity and that inhibition of MFAP4 is a feasible immunotherapeutic strategy for glioma. However, several limitations of this study should be acknowledged, and future research directions are proposed accordingly.

Despite the promising results from our bioinformatics analysis, several limitations should be noted. Firstly, the current study relies predominantly on transcriptomic data from public databases such as TCGA, GTEx, and GEO. The lack of experiments on the relevant regulatory mechanisms has led to the fact that we do not know the specific mechanism of action of MFAP4 in promoting the proliferation, migration and invasion of glioma cells. Future studies should combine *in vitro* and *in vivo* experiments to elucidate the mechanism of action of MFAP4’s biological functions in glioma cells. Secondly, while our analysis revealed significant correlations between MFAP4 levels and immune cell infiltration, these associations do not necessarily imply causation. Mechanistic studies are needed to explore how MFAP4 modulates the tumor microenvironment.

Future research should focus on addressing the following key questions. Firstly, conduct *in vitro* and *in vivo* experiments to elucidate the specific mechanism of MFAP4 in glioma cell proliferation, migration, and invasion. This will provide a deeper understanding of its biological significance in glioma progression. Secondly, investigate the mechanisms by which MFAP4 regulates ferroptosis. Our study found a positive correlation between MFAP4 levels and ferroptosis-related genes, but the underlying pathways remain unclear. Future research should explore how MFAP4 influences iron metabolism and lipid peroxidation to modulate ferroptosis. Lastly, assess the potential of MFAP4 as a therapeutic target. Although MFAP4 has been implicated in the prognosis of various cancers, its therapeutic potential remains underexplored. Evaluating the efficacy of antibodies or small-molecule inhibitors targeting MFAP4 in glioma treatment could provide new therapeutic strategies.

In conclusion, MFAP4 holds promise as a biomarker and potential therapeutic target in gliomas. Future studies should focus on elucidating the biofunctional mechanism of action of MFAP4, its role in ferroptosis, and its therapeutic potential. Addressing these questions will provide valuable insights and may lead to new diagnostic and therapeutic strategies for glioma patients.

## 5 Conclusion

Overall, our study provides the first evidence that MFAP4 is aberrantly overexpressed in gliomas and correlates with adverse clinicopathologic features. MFAP4 may serve as a valuable diagnostic and prognostic indicator. Given the integrative regulatory role of MFAP4 in tumor immunity as well as ferroptosis, therapeutic regimens that block MFAP4 may be a promising strategy for the development of more comprehensive and specific tumor immunotherapy for gliomas.

## Data Availability

The datasets presented in this study can be found in online repositories. The names of the repository/repositories and accession number(s) can be found in the article/Supplementary Material.
