# Supplementary figures and images for: MFAP4 is a novel prognostic biomarker in glioma correlating with immunotherapy resistance and ferroptosis
Source: Front Pharmacol. 2025 Feb 21;16:1551863. doi: 10.3389/fphar.2025.1551863 (PMC11885252; doi:10.3389/fphar.2025.1551863)

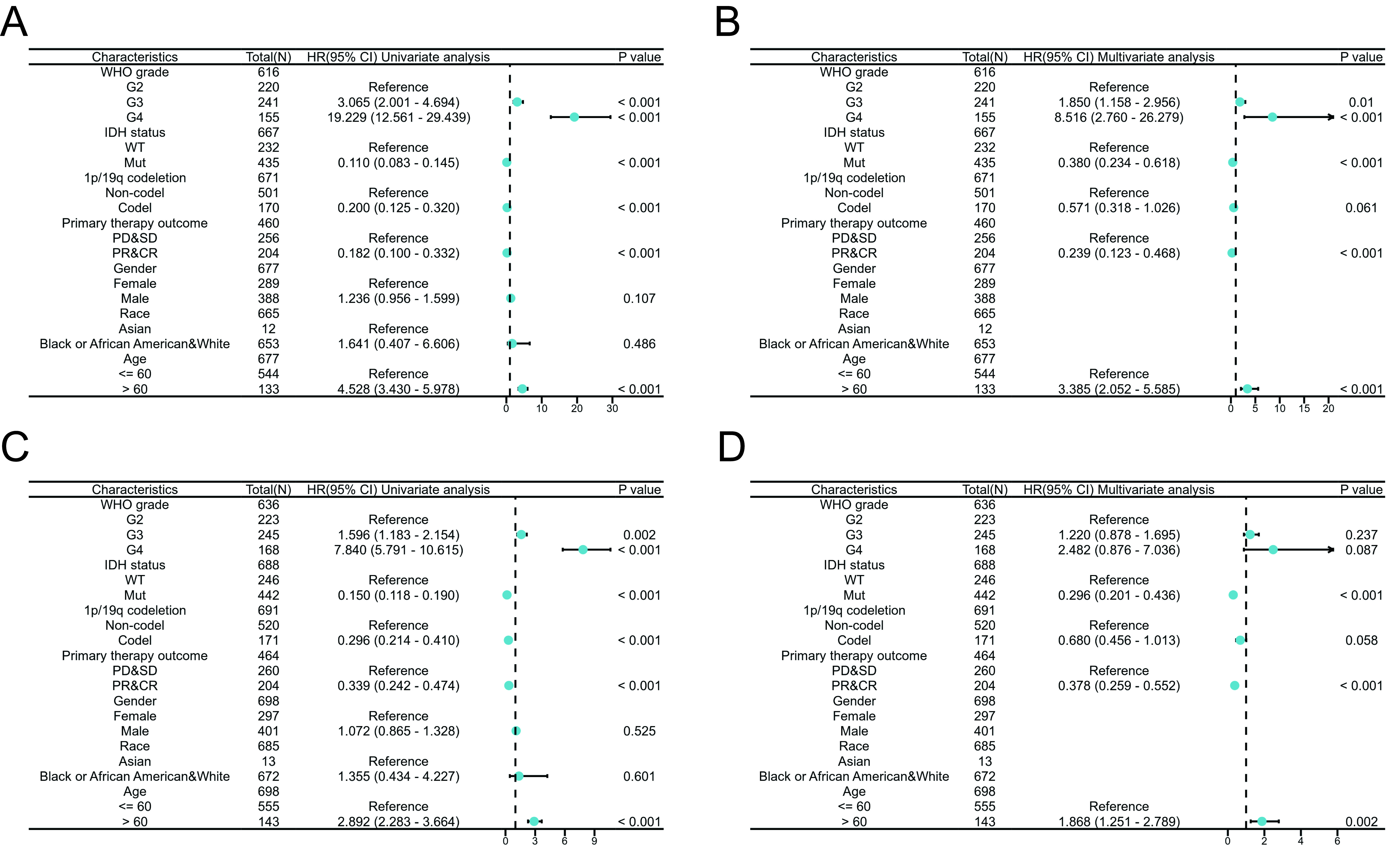

Supplement: Supplementary file 1 [file Image3.jpeg]

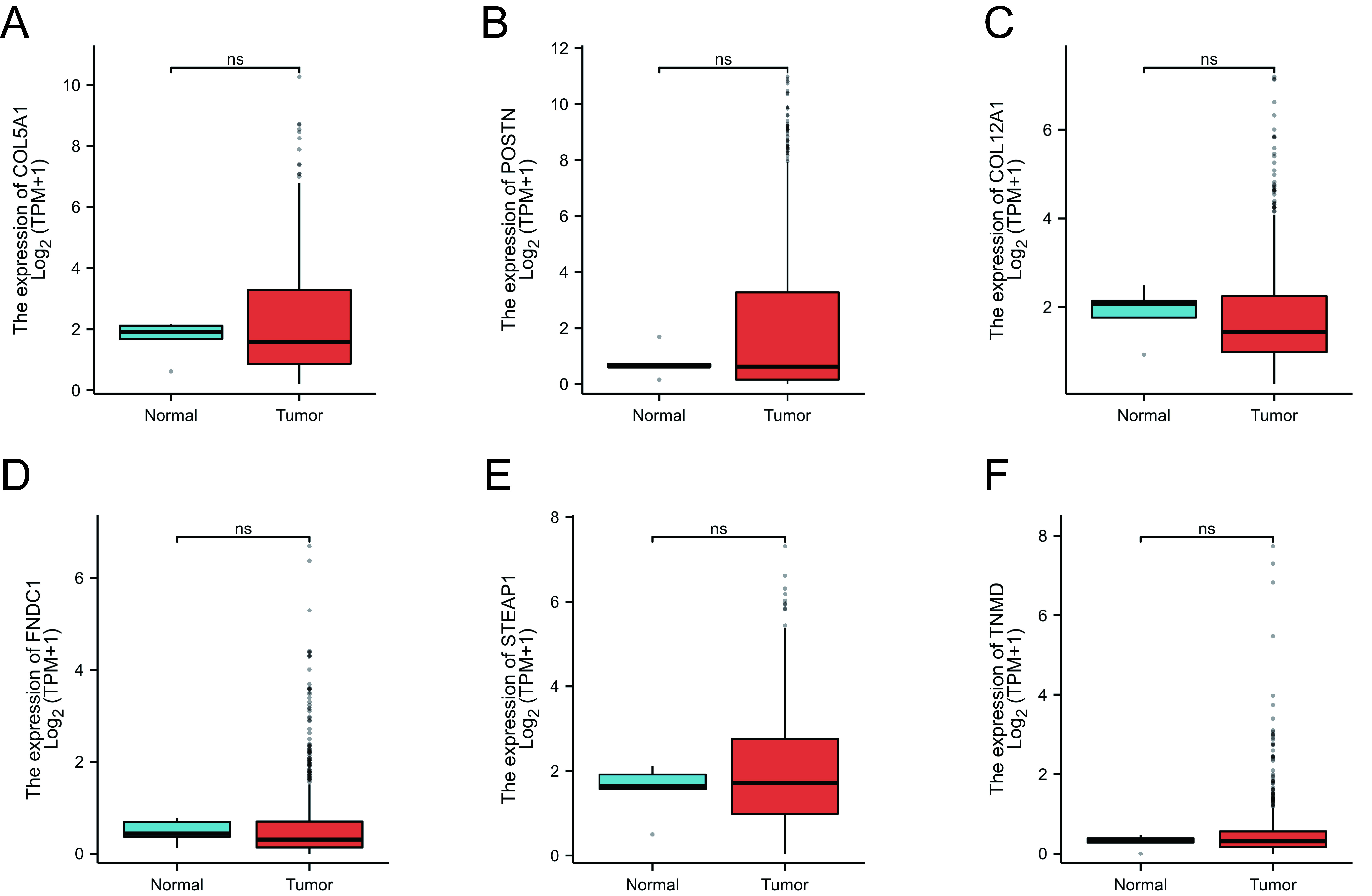

Supplement: Supplementary file 2 [file Image1.jpeg]

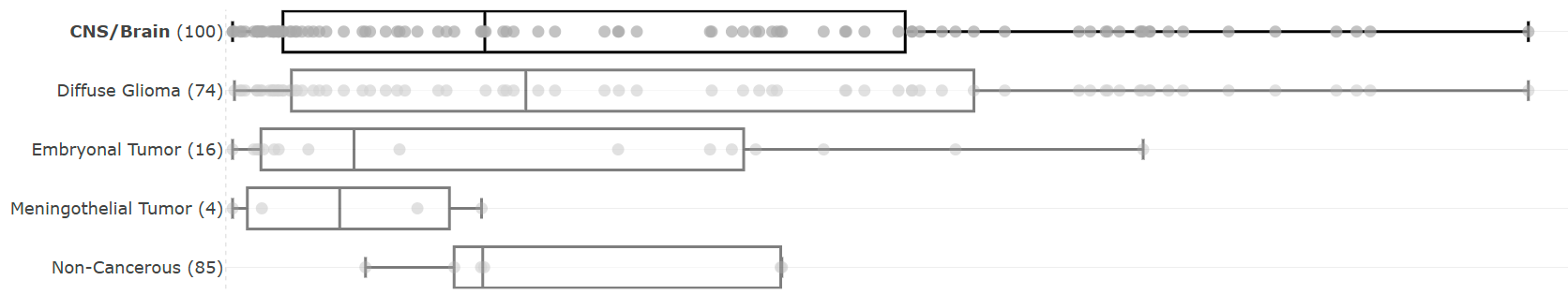

Supplement: Supplementary file 3 [file Image2.png]
